# Role of the inferior vena cava collapsibility index in predicting propofol-induced hypotension in patients undergoing colonoscopy

**DOI:** 10.1186/s12871-025-02945-y

**Published:** 2025-02-14

**Authors:** Zhou Zhou, Yujie Li, Jinxian Zhu, Yingge Liu, Yuxin Wang, Xiaoqiao Sang, Xinxin Wang, Xiaobao Zhang

**Affiliations:** 1 Department of Anesthesiology, The Affiliated Lianyungang Hospital of Xuzhou Medical University, Lianyungang, China; 2 Lianyungang Maternal and Child Health Hospital, Lianyungang, China; 3 Department of Anesthesiology, The Central Hospital of Wuhan, Tongji Medical College, Huazhong University of Science and Technology, Wuhan, China; 4 Department of Anesthesiology, Lianyungang Clinical College of Nanjing Medical University, Lianyungang, China; 5 Department of Anesthesiology, The First Affiliated Hospital of Kangda College of Nanjing Medical University, Lianyungang, China

**Keywords:** Colonoscopy, Inferior vena cava, Propofol-induced hypotension, Transthoracic echocardiography

## Abstract

**Background:**

Hypotension is a common side effect of propofol induction, and when severe, it is associated with adverse outcomes. Ultrasonography of the inferior vena cava (IVC) is a reliable indicator of the intravascular volume. This study investigated whether preoperative IVC ultrasound measurements could predict hypotension after propofol induction in patients undergoing colonoscopies.

**Methods:**

Sixty-two adult patients with American Society of Anesthesiologists physical status (ASA) I-II scheduled for colonoscopy after propofol induction were recruited. The Ultrasound Maximum IVC diameter (dIVCmax), minimum IVC diameter (dIVCmin), and collapsibility index (IVC-CI) were assessed in all patients before propofol induction. Mean blood pressure (MBP) was recorded before induction. Propofol was injected intravenously after ultrasound measurements. MBP was recorded 1, 3, 5, and 10 min after propofol induction. The receiver operating characteristic (ROC) curve of IVC-CI was compared with that of patients who developed hypotension after propofol induction.

**Results:**

Sixty-two patients completed the study, and their data were considered for statistical analysis. After induction,30 patients developed hypotension. The area under the curve (95% confidence interval) was 0.72 (0.595 to 0.849) for IVC-CI. The optimal IVC-CI cutoff value was 38.25%, with a sensitivity of 56.7% and specificity of 71.9%. IVC-CI before induction strongly correlated with the maximum percentage of MBP drop after propofol induction. (regression coefficient = 0.33, *P* = 0.008), respectively.

**Conclusion:**

Pre-induction IVC-CI > 38.25% is a non-invasive predictor of propofol-induced hypotension in patients undergoing colonoscopy and is strongly correlated with MBP drop.

**Trial registration:**

This clinical trial was approved by the Ethics Committee of The Affiliated Lianyungang Hospital of Xuzhou Medical University (YJ-20190529001). All the study procedures were performed in accordance with the ethical standards of the Helsinki Declaration of 2013.

## Introduction

Propofol is an optimal intravenous anesthetic commonly used for the induction and maintenance of anesthesia [1]. Propofol is a highly efficacious anesthetic with a rapid onset of action and relatively short recovery time. These attributes have led to their selection for anesthesia induction during colonoscopy. In addition, propofol exerts a profound sedative effect and is capable of alleviating anxiety in patients undergoing colonoscopy [2, 3]. Nevertheless, the cardio-vasodilatory effect of propofol may result in hemodynamic instability following induction of hypotension, which is a common adverse event. Potential mechanisms for this include decreased sympathetic tone, reduced preload, and afterload or direct myocardial depression [4]. A previous study showed that the incidence of hypotension following propofol induction was as high as 70% [5].

Hypovolemia is the most likely risk factor for postinduction hypotension; therefore, the identification and management of latent hypovolemia can reduce the incidence of such complications [6]. Although patients with normal cardiorespiratory function and stable hemodynamics can tolerate this side effect, in patients undergoing colonoscopy, preexisting hypovolemia due to dehydration, preoperative fasting and water restriction, bowel preparation, and impaired compensatory responses can exacerbate hypoperfusion of vital organs, which can increase the incidence of propofol-induced hypotension [7, 8]. It is unclear whether a transient decrease in blood pressure following propofol induction affects patient prognosis. However, studies have demonstrated that severe hypotension after induction can result in inadequate organ perfusion and the emergence of adverse postoperative outcomes, including myocardial infarction, acute kidney injury, heart failure, stroke, and prolonged hospitalization with elevated postoperative mortality [9, 10].

Transthoracic echocardiography (TTE) is an important tool for noninvasive hemodynamic monitoring. Measurement of the inferior vena cava (IVC) by TTE provides a cross-sectional image for rapid assessment of intravascular volume status and is commonly used in intensive care units (ICUs) and emergency departments [11]. Several studies have demonstrated that ultrasound measurement of the IVC collapsibility index (IVC-CI) and diameter can be used to assess volume status in perioperative patients [12, 13]. Measurement of the IVC using ultrasound before general anesthesia has been reported to predict hypotension after induction [6, 10]. In 2019, Arican et al. reported a predictive relationship between IVC-CI and fentanyl and propofol-induced hypotension during colonoscopy [14]. Patients with a lower intravascular volume may be more prone to post-induction hypotension (PIH); however, this has not yet been explored. However, in most of these previous studies, combination regimens such as general anesthesia or propofol combined with fentanyl and the simultaneous action of different drugs on the vasculature and heart can complicate the mechanism of PIH. These factors may complicate the predictive value of IVC ultrasonography for PIH. Therefore, this study aimed to assess the predictive value of lower intravascular volume status measured using IVC ultrasonography for propofol-induced hypotension in patients undergoing colonoscopy without other drug side effects.

## Materials and methods

### Patients

This study was approved by the Ethics Committee of the Affiliated Lianyungang Hospital of Xuzhou Medical University (YJ-20190529001). This study was registered in the Chinese Clinical Trial Registry (ChiCTR1900027052). Before commencement of the study, written informed consent was obtained from all eligible patients. All the study procedures were performed in accordance with the ethical standards of the Helsinki Declaration of 2013.

The inclusion criteria were patients aged between 18 and 65 years without sex restriction and scheduled for colonoscopy, with ASA grades I or II. Before undergoing colonoscopy, patients were required to undergo a basic physical examination. Electrocardiography was required for patients over 40 years of age.

Patients with significant peripheral vascular disease, severe heart valve disease, unstable angina, an ejection fraction < 40%, respiratory distress, increased intra-abdominal pressure, autonomic nervous system disorders, an implanted pacemaker or cardioverter, a mental illness, an emotional or intellectual disability, or who declined to sign the informed consent form were excluded. Patients requiring endoscopic therapy or exhibiting poor IVC visualization, those currently taking angiotensin-converting enzyme inhibitors or angiotensin receptor blockers, and those who underwent colonoscopy for more than 15 min were also excluded. Before commencement of the study, written informed consent was obtained from all eligible patients.

### Outcomes

Primary outcome is the correlation of IVC-CI with the occurrence of propofol-induced hypotension during colonoscopy. Secondary outcome is the dIVCmin and dIVCmax.

### IVC ultrasonography

**Fig. 1:**
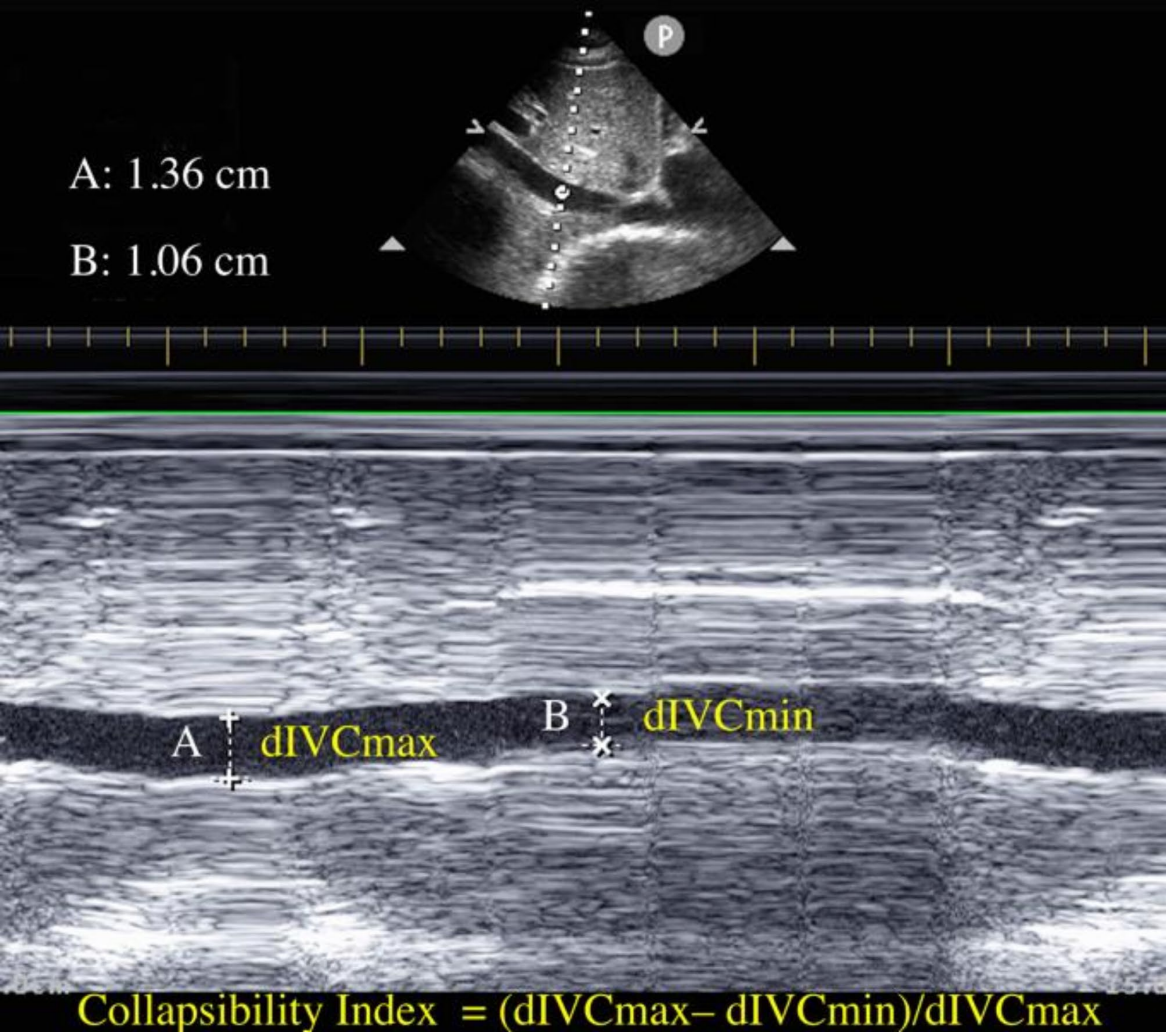
Ultrasound measurements of inferior vena cava (IVC) and calculation of collapsibility index. Panel above shows two-dimensional scan of the IVC, and panel below shows M-mode scan with respiratory variations in diameter. dIVCmax = maximum diameter of IVC; dIVCmax = minimum diameter of IVC

The IVC-CI was measured before induction of anesthesia. The IVC-CI was assessed by the same trained anesthesiologist who was blinded to the postinduction hemodynamic measurements. All patients were conscious, supine, and spontaneously breathed for at least 5 min before the IVC examination. An M-mode phased-array transducer (3.5 MHz) of transthoracic echocardiography (Philips CX50; Bothell, WA, USA) was used to measure the IVC diameter and its variation. A micro-convex array transducer was used to identify and measure the maximum diameter of the IVC (dIVCmax) and minimum diameter of the IVC (dIVCmin) during spontaneous breathing, after which the sagittal section of the IVC at the subxiphoid position was scanned. The operator must have a fulcrum on the patient’s skin to ensure that the sonologist remains on the axis. A distance of 2 cm from the junction point of the right atrium and IVC was selected to obtain images of dIVCmax and dIVCmin (including 2–3 breathing cycles). dIVCmax was measured at the end of expiration, whereas dIVCmin was measured at the end of inspiration. Three scans were performed for each patient to ensure the reliability of the IVC measurements. If there was a difference of more than 0.2 cm in the dIVCmax measurements between any two images, the patient’s data were excluded from the study [10, 14]. The entire IVC scan procedure was completed within 10 min. The image deemed to be of highest quality was selected for each patient. The maximum and minimum IVC diameters were measured over a single respiratory cycle using built-in software. CI was calculated as CI = (dIVCmax– dIVCmin)/dIVCmax (Fig. 1) and expressed as a percentage [15, 16]. All hemodynamic and ultrasonographic data were collected by an experienced anesthetist with basic experience in echocardiography who had previously undergone more than 20 IVC ultrasound examinations.

### Preoperative preparation for colonoscopy

Patients undergoing colonoscopy were required to undergo an 8-hour preoperative fasting from food and then fast from water for 4 h. Additionally, the patients were required to undergo bowel preparation before colonoscopy. It is recommended that patients commence taking compound polyethylene glycol electrolyte powder (PEG) 4–6 h before colonoscopy and finish it within 2 h. If bowel movements fail to meet these criteria, a PEG solution or water may be added, but the total volume should not exceed 4 L. In patients with risk factors for inadequate bowel preparation (e.g. chronic constipation, depression, etc.), a split-dose regimen of PEG may be used to prepare the bowel. The split-dose PEG regimen is routinely used as a 3 L PEG regimen in divided doses, comprising 1 L administered 10–12 h prior to the scheduled bowel examination and 2 L administered 4–6 h prior to the examination on the day of the examination.

### Anesthesia management

Upon entering the endoscopy room, vital signs of the patients were recorded. Electrocardiograms, blood pressure (BP) readings, and peripheral oximeter measurements were obtained for all patients. BP was measured using a non-invasive oscillometric cuff. Oxygen was administered via nasal cannula at a flow rate of 4 L/min. Blood pressure was measured using a cuff on three occasions in a resting state, and the readings were averaged to establish the baseline blood pressure. TTE was used to ascertain dIVCmax and dIVCmin. Subsequently, propofol (2.0–3.0 mg/kg) was administered intravenously at a rate of 10 mg every 5 s. The objective of propofol titration for induction is to ensure that the patient exhibits no response to painful stimulation (e.g., squeezing the trapezius). Changes in MBP were measured 1, 3, 5, and 10 min after propofol induction with a cuff. Severe (MBP < 55 mmHg) or prolonged (duration greater than or equal to 2 min) episodes of hypotension were treated with intravenous boluses of norepinephrine (8 ug), if accompanied by a heart rate of less than 50 beats per minute, intravenous boluses of ephedrine (3 mg). A heart rate (HR) < 45 bpm was treated with an intravenous bolus of atropine (0.5 mg).

### Data collection

The following demographic data were recorded: age, sex, height, weight, and hypertension history. The current medications used to treat hypertension were also recorded. The average of three MBP readings obtained prior to induction was designated as the baseline. Non-invasive BP and HR were recorded before induction. Non-invasive BP was recorded at 1, 3, 5, and 10 min after propofol induction. The maximum percentage change relative to the baseline value was calculated by using the lowest MBP value after propofol induction. The term “hypotension” was defined as a 20% reduction in MBP or an absolute MBP value < 65 mmHg [17]. Similarly, “severe hypotension” was defined as an MBP value < 55 mmHg [18].

### Statistical analysis

#### Sample size

A review of data from previous relevant studies revealed that IVC-CI had a sensitivity of 78.6% and specificity of 91.7% for predicting the occurrence of post-induction hypotension [19]. A sample size calculation was conducted using the PASS software with a significance level of 0.05, a tolerance error of 0.1, and a two-sided test. This yielded a sample size of 60, which required the inclusion of 60 patients for the analysis.

#### Data analysis

SPSS version 26.0 (IBM Corporation, Armonk, New, USA York) was used for all statistical analyses. Data collected during the study were compiled using Excel spreadsheets (Microsoft, USA). The normality of the data was tested using the Kolmogorov–Smirnov one-sample test. The data are presented as mean ± standard deviation (SD) for continuous variables and as absolute numbers or percentages for categorical variables. Non-normally distributed measurement data were expressed as medians and interquartile ranges.

The development of clinically significant hypotension after induction was analyzed with respect to patient characteristics, hemodynamic data, and IVC measurements using Student’s t-test or the χ2 test, where appropriate. IVC-CI values were correlated with the percentage of maximum drop in MBP values from baseline after induction of propofol (Pearson correlation). The diagnostic performance of the test was evaluated by examining the specificity, sensitivity, and area under the receiver operating characteristic (ROC) curve. ROC curve analysis was performed to ascertain the capacity of the ultrasound-derived parameter IVC-CI to predict clinically significant hypotension after propofol induction across the entire cohort. AUCs were calculated along with their 95% confidence intervals. The optimal cutoff values were identified as those that maximized the Youden index (sensitivity + specificity − 1) [20]. Sensitivity and specificity with 95% confidence intervals for the optimal cutoff values are presented. A two-tailed *p*-value of less than 0.05 (two-tailed) was considered statistically significant.

## Results

**Fig. 2:**
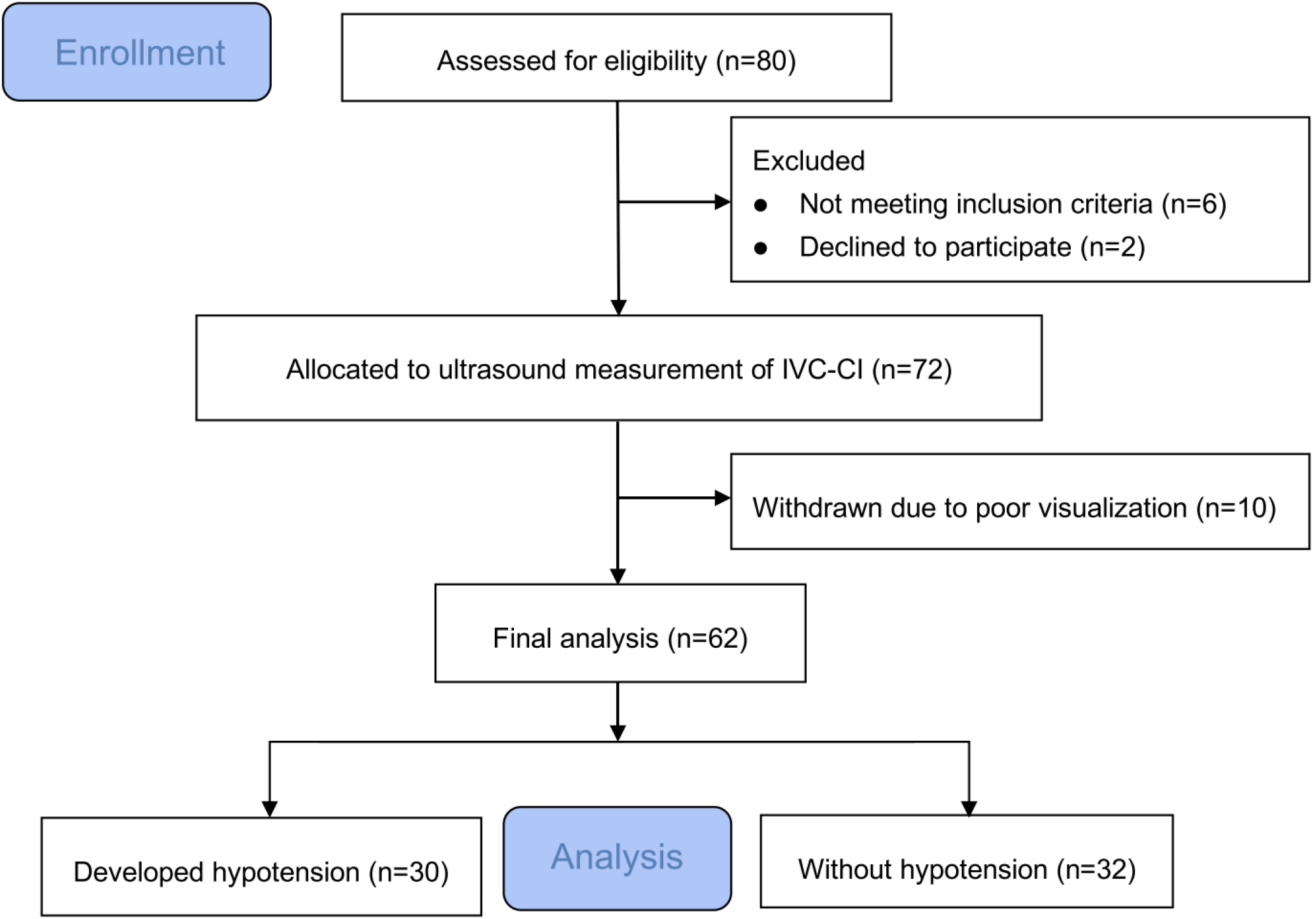
Flowchart of patients included in the present study

Of the 80 eligible patients, six were excluded due to treatment with ACEIs or ARBs, two refused to participate in the study, and another ten patients (13.9%) were excluded due to poor IVC visualization (Fig. 2). Sixty-two patients completed the study, and their data were considered for statistical analysis Among these, 24 patients had a history of hypertension (Table 1), 10 were taking β-blockers, six were on calcium channel blockers, three were on thiazide diuretics, and three were not on medications.

**Table 1:** Comparison of patient characteristics, hemodynamic data, and preoperative inferior vena cava (IVC) ultrasound measurements between patients who did and did not develop hypotension after propofol induction

	Developed Hypotension		
Variable	Yes (*n* = 30)	No (*n* = 32)	*P* Value
Age, year	46.40 ± 14.36	41.78 ± 14.48	0.213
Sex (male/female)	16/14	16/16	0.793
BMI, kg/m^2^	23.75 ± 3.79	24.00 ± 3.55	0.782
ASA (I/II)	16/14	11/21	0.132
Baseline HR, beats/min	77.63 ± 10.11	73.44 ± 13.39	0.171
Baseline MBP, mmHg	94.70 ± 12.89	92.97 ± 13.98	0.615
Propofol dose, mg/kg	2.58 ± 0.22	2.47 ± 0.28	0.097
IVC-CI (%)	41.02 ± 12.07	32.34 ± 9.39	0.003
dIVC_min_, cm	0.87 ± 0.37	0.98 ± 0.33	0.252
dIVC_max_, cm	1.45 ± 0.44	1.44 ± 0.45	0.943
History of hypertension (yes/no)	12/18	12/20	0.840

Data are expressed as mean ± SD or absolute number (percentage). ASA = American Society of anesthesiologists physical status; BMI = body mass index; HR = heart rate; MBP = mean blood pressure; IVC-CI = collapsibility index of IVC; dIVCmax = maximum diameter of IVC; dIVCmax = minimum diameter of IVC

### Hemodynamic data

**Fig. 3:**
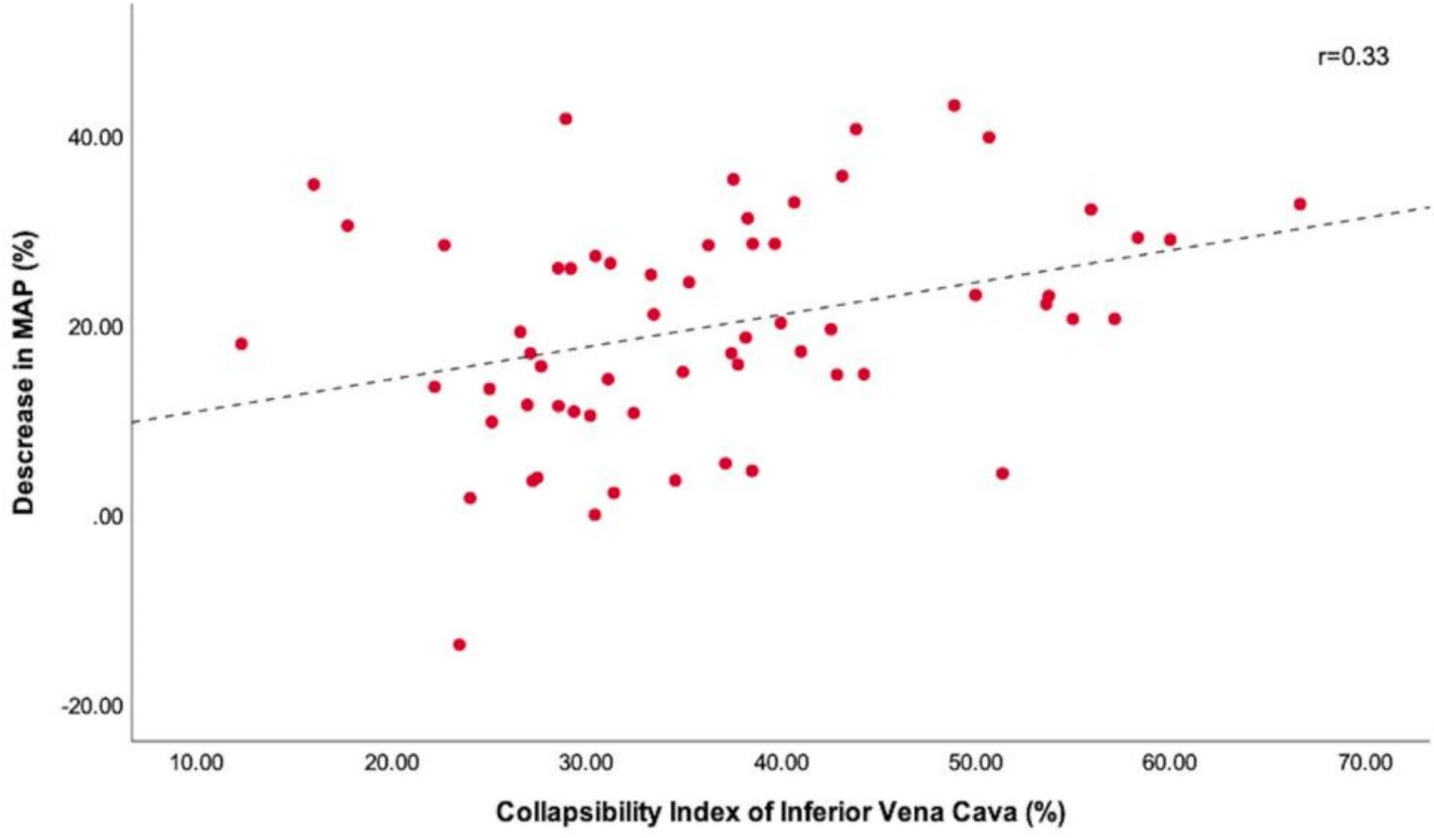
Scatter plots showing the relationships of collapsibility index of inferior vena cava with percentage decrease in mean blood pressure from baseline after propofol induction. Trend lines are represented by dotted lines; regression coefficient = 0.33. MBP = mean blood pressure; r = regression coefficient

After induction of propofol, 30 (48.7%) patients exhibited a reduction in blood pressure in accordance with the study criteria. Of these, 16 patients exhibited an MBP of less than 65 mmHg. Three patients received noradrenaline and three received ephedrine for severe hypotension lasting more than two minutes. One patient received atropine for bradycardia. There were no significant differences in baseline MBP and HR between patients who developed hypotension and those with more stable blood pressures. Greater IVC-CI was observed in patients who developed hypotension (*P* < 0.05, Table 1). Increased IVC-CI positively correlated with decreased MBP (*r* = 0.33; *P* = 0.008; Fig. 3). No significant difference was observed in the dose of propofol administered to the patients with and without hypotension (*P* > 0.05, Table 1). A weak association was observed between a decrease in MBP after induction and IVC measurements. The dIVCmin and dIVCmax were not statistically different between the two groups.

### ROC curve analysis for all patients

**Fig. 4:**
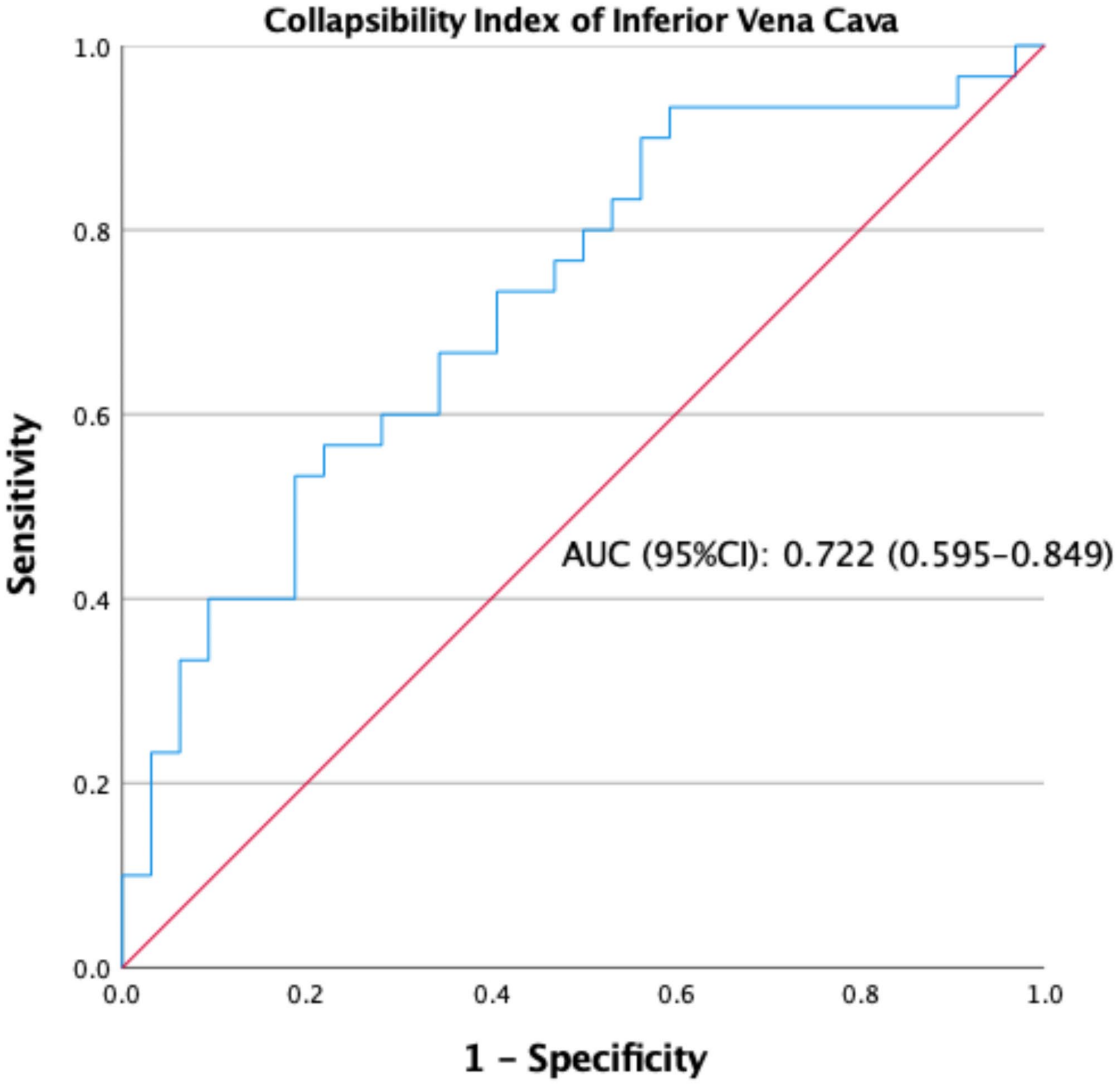
Receiver operating characteristic curves showing the ability of collapsibility index of inferior vena cava to predict hypotension after propofol induction

ROC curve analysis for prediction of hypotension following propofol induction. The optimal cutoff value of IVC-CI was 38.25%, and IVC-CI had an AUC of 0.722 under the ROC curve (95% CI: 0.595–0.849, Table 2). Sensitivity and specificity were 56.7% and 79.1%, respectively (Fig. 4).

**Table 2:** The performance of IVC-CI as a predictor of hypotension after induction of propofol in patients undergoing colonoscopy

Cut off	AUC (95%CI)	Sens. (%)	Spec. (%)	*P* value
> 0.38	0.722 (0.595–0.849)	56.70%	79.10%	< 0.05

IVC-CI: Inferior vena cava collapsibility index, AUC: Area under curve, CI: Confidence interval, Sens: Sensitivity, Spec: Specificity

## Discussions

In patients undergoing colonoscopy after receiving propofol for induction of anesthesia, a pre-induction inferior vena cava collapsibility index > 38.25% can be a simple, non-invasive, and reliable predictor of post-induction hypotension with propofol. IVC-CI before induction strongly correlated with the maximum percentage of MBP drop after propofol induction. After propofol induction, 30 patients (48.7%) exhibited a reduction in blood pressure according to the study criteria.

It has been demonstrated that the IVC is a highly compliant volumetric vessel and that its diameter is a reliable indicator of volume status [21–25]. There is a strong correlation between this diameter and the right atrial pressure and blood volume. Respiratory changes can be utilized to predict fluid responsiveness [26]. During inspiration, a decrease in intrathoracic pressure results in an increase in blood flow from the IVC back to the right atrium, which subsequently leads to a decrease in IVC diameter. Conversely, a decrease in intrathoracic pressure during expiration results in an increase in IVC diameter. A larger IVC-CI is indicative of a hypovolemic state, particularly in the presence of a smaller IVC diameter. The current literature suggests that effective volume resuscitation is associated with a reduction in IVC-CI [27]. IVC diameter is positively correlated with central venous pressure (CVP), whereas IVC-CI is negatively correlated with CVP [28]. The American Society of Echocardiography guidelines recommend the use of ultrasonography to measure the IVC and assess a patient’s volume status [26]. Numerous studies have shown that IVC-CI assesses volume status and is a reliable predictor in patients at a high risk of hypotension [26, 29, 30].

Currently, propofol is one of the most frequently used anesthetic agents for colonoscopies. Nevertheless, propofol is also associated with a few side effects including decreased systemic vascular resistance, sympathetic depression, and decreased cardiac output [31]. The prolonged duration of colonoscopy may necessitate the administration of higher doses of propofol to induce anesthesia. In a Meta-analysis summarizing data from 6 clinical trials of patients undergoing colonoscopy, hypotension was more common with propofol sedation, with an incidence of 35%. Longer propofol sedation and higher propofol doses result in hypotension last longer and more severe hypotension, thus reducing the quality of postoperative recovery and prolonging postoperative recovery time [32]. Therefore, it is of great importance to be able to recognize and predict hypotension after propofol induction at an early stage so that we can intervene in advance to reduce the incidence of hypotension. Therefore, the present study sought to examine the potential of changes in IVC diameter and CI in predicting the occurrence of hypotension after propofol induction.

In this study, there was no significant difference in the maximum and minimum diameters of the veins between patients who developed hypotension and those with a more stable blood pressure. This may be because the absolute value of the diameter of large veins varies considerably between individuals to indicate blood volume status. However, changes in the diameter of large veins reflect not only blood volume status but also blood volume responsiveness. The main reason for propofol-induced hypotension is the relative insufficiency of circulating blood volume due to vasodilatation [33]. This does not necessarily imply absolute volume deficiency. Therefore, there may be no significant difference in absolute IVC diameter.

The use of ultrasonography to evaluate the IVC before induction may assist in identifying of patients who are likely to experience hypotension. Furthermore, this technique can be employed by healthcare providers to inform decisions regarding sedation, propofol dosage, and administration of prophylactic intravenous fluids. Volume assessment represents a fundamental aspect of perioperative therapy in which a range of subsequent treatments is based. Accurate assessment of intravascular volume status and responsiveness can ensure adequate tissue perfusion and improve prognosis. Prolonged fasting and water restriction before colonoscopy can result in volume deprivation, whereas gastrointestinal preparation can further exacerbate hypovolemia. In addition, propofol administration results in vasodilation and myocardial depression, thereby increasing the likelihood of severe hypotension. Therefore, effective blood volume monitoring indicators must be used to predict whether propofol-induced hypotension occurs in patients undergoing colonoscopy. Previous studies have demonstrated that IVC-CI is a non-invasive, simple, rapid, and reliable indicator of intravascular volume status [34, 35]. This study aimed to identify the critical value of IVC-CI by using ROC curves for the prediction of hypotension following propofol-induced hypotension.

A multicenter study showed, ultrasound scanning of the IVC and measurement of the.

IVC-CI preoperatively provides a reliable predictor of hypotension after the induction of general anesthesia in 75% of patients. In patients at a high risk of complications resulting from intraoperative hypovolemia and hypotension, measurement of IVC-CI may provide clinically useful information [10]. Muller et al. showed that patients with IVC-CI ≥ 40% exhibited a heightened risk of developing hypotension following propofol induction. The results of our study indicated that the threshold for predicting propofol-induced hypotension was 38.25%, which was less than the results of the study by Muller et al. Upon analysis of the two studies, it can be postulated that the discrepancy may be attributed to the differing definitions of hypotension [36]. Although intraoperative hypotension is a common side effect of anesthesia, the definition of this phenomenon varies among different clinical studies. Bijker et al. found 140 definitions of hypotension in the literature, which resulted in a wide range of reported incidences [37]. Although there are multiple thresholds defining intraoperative hypotension, two thresholds associated with the prognosis of postoperative myocardial and renal injuries were selected for this study. A drop in blood pressure of > 20% from baseline or a blood pressure of less than 65 mmHg was selected as the definition of hypotension. A previous study also demonstrated that preoperative ultrasound IVC measurements revealed that patients with IVC-CI of greater than 43% were more likely to be hypotensive after the induction of general anesthesia [10]. One distinction is that the distinction of anesthesia was performed with etomidate rather than propofol. Furthermore, the hemodynamic stability of etomidate is well documented. However, propofol is known to cause hypotension following induction owing to vasodilation and myocardial depression [38].

Patients who underwent colonoscopy were selected for this study. Preoperative fasting and bowel preparation had a sufficient effect on the volume of these patients to mimic the status of hypovolemic patients rather than selecting patients with a very poor systemic status. Second, only propofol was administered to patients undergoing colonoscopy at the time of induction, which differs from hypotension induced by intravenous combined anesthesia. In this manner, the interaction with other drugs Is reduced, thereby allowing for a more acute reflection of the occurrence of hypotension following propofol induction. Further experiments could also investigate the incidence of propofol-induced hypotension in patients undergoing colonoscopy after a rapid intravenous infusion strategy before anesthesia induction and further validate the diagnostic value of ultrasound-guided vena cava measurements for PIH with propofol.

This study has the following limitations: First, it included only patients with ASA classes I and II. These patients typically had a good baseline conditions and stable circulatory compensation. In addition, patients with a history of severe respiratory and cardiac diseases were excluded from this study; therefore, the results do not apply to patients with more severe conditions. Second, patients taking ACEIs, or ARBs medications were excluded from the study to avoid their high vasodilatory effect as a confounder; therefore, the results of the current study cannot be applied to such patient populations. Third, ultrasonographic IVC measurements were performed by an operator with basic echocardiographic experience. Nevertheless, the reliability of ultrasound inferior vena cava assessment is not contingent on the operator’s level of echocardiographic experience. It was not within the scope of this study to assess the accuracy of inferior vena cava measurements. A previous study demonstrated moderate interrater reliability of IVC measurements [39]. In addition, the IVC was scanned three times for each patient, and the difference between dIVCmax readings was consistently less than 0.2 cm. Fourth, blood pressure was measured using only the non-invasive oscillometric method, which has a defined time interval for data collection. This may result in the omission of some instances of hypotension, potentially leading to statistical error.

## Conclusions

An inferior vena cava collapsibility index > 38.25% before anesthetic induction can be a simple, noninvasive, and reliable predictor of hypotension after propofol induction in patients undergoing colonoscopy. IVC-CI was positively correlated with the maximum decrease in MBP after propofol induction and can be used to guide aspects of preoperative preparation and anesthetic management. Clinically, preoperative IVC-CI measurements are easy and rapid to obtain, and IVC ultrasonography can be integrated into routine clinical practice. However, the study only included patients in good baseline condition, who usually have stable circulatory compensation. Further research is needed to validate these findings in larger, more diverse populations. Future research based on CI measurements is needed to determine the best intravenous fluid strategy to reduce postinduction hypotension.

## Data Availability

No datasets were generated or analysed during the current study.

## Declarations

### Writing process

Authors did not use AI in the writing process of this research.

## Abbreviations

IVC: Inferior vena cava
IVC-CI: Inferior vena cava collapsibility index
MBP: Mean blood pressure
dIVC: Diameter of the inferior vena cava
dIVCmax: Maximum IVC diameter
dIVCmin: Minimum IVC diameter
ASA: American Society of Anesthesiologists physical status
TTE: Transthoracic echocardiography
ICUs: Intensive care units
ACEIs: Angiotensin-converting enzyme inhibitors
ARBs: Angiotensin receptor blockers
PIH: Post-induction hypotension
PEG: Polyethylene glycol electrolyte powder
BP: Blood pressure
HR: Heart rate
CVP: Central venous pressure
ROC: Receiver operating characteristic
AUC: Area under curve
